# Supplementary material for: Learning causal networks using inducible transcription factors and transcriptome‐wide time series
Source: Mol Syst Biol. 2020 Mar 17;16(3):e9174. doi: 10.15252/msb.20199174 (PMC7076914; doi:10.15252/msb.20199174)
Supplement: Supplementary file 1 — Appendix [file MSB-16-e9174-s001.pdf]

Appendix
Learning causal networks using inducible transcription factors and transcriptome-wide time series

Sean R. Hackett[1], Edward A. Baltz[2], Marc Coram[2], Bernd J. Wranik[1], Griffin Kim[1], Adam Baker[1], Minjie Fan[2], David G. Hendrickson[1], Marc Berndl[2], R. Scott McIsaac[1]

[1]Calico Life Sciences LLC, 1170 Veterans Blvd., South San Francisco, CA 94080, USA.
[2]Google Research, 1600 Amphitheatre Parkway, Mountain View, CA 94043, USA.

Contents

1 Obtaining the *noise-value thresholded* gene expression dataset 3
2 Modeling timecourses with sigmoidal or impulse-like dynamics 4
3 Obtaining the *shrunken* gene expression dataset 5
4 Obtaining kinetic parameters for timecourses 5
5 Dynamical systems modeling 6
6 Linear Regression 8
7 BIC regularization 9
8 Hyperparameters 11
9 Model Validation by Holdout 11
10 Validation Experiments 12
11 **Appendix Figure S1:** Gene expression responses do not depend on triggering mechanism and TF induction is rapid across our dataset 14
12 **Appendix Figure S2:** Estimating leakiness and inducible of synthetic promoter-driven TF alleles 15
13 **Appendix Figure S3:** Induced changes can be robust to environmental state 16
14 **Appendix Figure S4:** Systematic identification and summarization of regulatory signals 17
15 **Appendix Figure S5:** Full transcriptome of the “raw” gene expression data 18
16 **Appendix Figure S6:** Full transcriptome of the “shrunken” gene expression data 19
17 **Appendix Figure S7:** Counts of impulse vs. sigmoids across experiments 20
18 **Appendix Figure S8:** Most impulses exhibit near-perfect adaptation. v_{inter} is compared to v_{final} for all timecourses exhibiting impulse dynamics 21
19 **Appendix Figure S9:** Diverse, functionally significant regulation in the Pho4 induction experiment 22
20 **Appendix Figure S10:** Functional classes of kinetic responses 23
21 **Appendix Figure S11:** Response magnitude comparison with reported regulation 24
22 **Appendix Figure S12:** Extent of differential expression per experiment or gene 25
23 **Appendix Figure S13:** Histograms of maximum correlation of genes to all other measured genes are shown as experiments are pruned from the dataset 26
24 **Appendix Figure S14:** Fit of regulatory model to observed gene-expression measurements 27
25 **Appendix Figure S15:** Negative genetic interactions with rpn4Δ from http://thecellmap.org 28
26 **Appendix Figure S16:** Fit of regulatory model to observed gene-expression measurements 29
27 **Appendix Figure S17:** Transcriptome-wide heatmap for 10 validation experiments 30
28 **Appendix Figure S18:** Identifying latent transcriptional regulatory hubs 31
29 **Appendix Figure S19:** GRNs 32
30 **Appendix Figure S20:** Comparing transcriptome data from two Fmp48 overexpression datasets 33

1 Obtaining the *noise-value thresholded* gene expression dataset

Biological and technical variability limit the precision of any measurement of gene expression. Under the assumption that any gene is not affected in most experiments, the width of the central quintile (20%) of "cleaned" log-ratio measurements is an accurate estimate of the total noise level for that gene. We assumed log-normal distributions for each gene, mapping central quintile to standard deviations (central quintile = $\pm 0.253\sigma$ for normal). The typical standard deviation (i.e., noise estimate) for cleaned log-ratios (base 2) is approximately 0.1 for genes with green medians above about 200. Genes with lower green medians have higher noise levels, up to about 0.75 (a factor of 1.7).

We then compared this gene-level noise estimate with the typical (median) spot-to-spot error for the genes. We found that the two are mostly uncorrelated, with the spot-to-spot error being significantly smaller. For genes with low green median, the spot-to-spot error accounts for most of the gene error.

Given a noise model, we can now consider which data points represent credible discoveries. We first considered each gene separately, and compare the cleaned log-ratio to the "universal threshold", as defined in [1]. This approach uses the fact that the maximum of N draws from a normal distribution is close to $\sqrt{2 \ln N}\sigma$. If the bulk of the noise is Gaussian, we can control the probability of at least one false positive. For the 1,500 non-zero time points for a given gene, this corresponds to 3.8σ. We flagged all measurements that exceeded this threshold in either direction (a total of 3.3% of the time points). The first level of thresholding is to set all timecourses with no points passing threshold to exactly zero.

Noise levels can also vary between microarrays. Thus, we zeroed out any timecourse that might have signal according to the gene-only noise model, and then computed the microarray-level noise from the central quintile of the genes that were not filtered out. We then constructed a simple metric to describe undesirable timecourses. Timecourses with a single significant detection (not at the final time point), or timecourses with non-consecutive significant detections, are not likely to be true positives. We then chose a weighting factor to minimize the fraction of detected timecourses that are of these types [noise model: $\sigma^2_{total} = (5/6)\sigma^2_{gene} + (1/6)\sigma^2_{microarray}$]. This removed obvious microarray-level artifacts while preserving the signal in experiments where a large fraction of the genome is affected.

Collectively, the full cleaned dataset is 1,693 microarrays in 217 experiments, each with 6,175 genes. Of the 1.34 million time series, 118,134 (8.8%) have at least one point that passes the full noise model. 60,968 (4.5%) have multiple points that pass the noise model. Of the time series with only single detection, about half occur in the final time point, suggesting that these are genuine late changes. Conversely, 9,329 time series possessed gaps between detections suggesting that their changes may be non-biological. These two filters (removing non-final singletons and non-consecutive changes) left 79,709 timecourses (5.9%),

which were used to train the whole-cell dynamical systems model.

2 Modeling timecourses with sigmoidal or impulse-like dynamics

Next, we wanted to identify timecourses that most likely contain smooth, biologically-feasible dynamics. In order to capture dynamics without overly restricting the types of dynamics that might exist, each timecourse with observation-level signal was fit with the phenomonological impulse model of Chechik and Koller [2]. This model expresses timecourses as the sum of two sigmoidal changes each which have a characteristic amplitude and time constant. This impulse model has been sufficient to capture complex pattern in expression and metabolomic data. timecourses were considered well fit by an impulse model if over 80% of expression variation could be accounted for through the impulse fit (i.e., $\sum (\hat{f}_{ij} - f_{ij})^2 / \sum f_{ij}^2 < 0.2$).

This filter removed highly inconsistent responses, but in some cases dynamics were clearly driven by an outlier yet an impulse model still fit the data well. To remove these cases, two heuristics were applied to separately remove outliers driven by the time zero measurement, and those occurring at an intermediate time point. In the first few minutes of an induction experiment, few strong transcriptional changes exist besides the induced transcription factor. In practice if the time zero point was noisy, measurements that are normalized together with respect to this value will be systematically higher or lower than expected, resulting in sharp early change in expression. Such changes were removed by filtering timecourses where the largest absolute change in expression was between the first and second timepoint and in which the impulse model explained less than 98% of variation is explained by the impulse model (the latter filter recovered early, strong changes such as the primary induction event). The second case of pathological timecourses were cases where a single outlier measurement (beyond what could be detected by our Gaussian noise model) was not appropriately reflected in either the preceding f_{t-1}, nor the succeeding f_{t+1} time point despite fine temporal samples. Large changes followed by a return to baseline were distinguished from continuous timecourse responses by removing timecourses with a serial sum of squares to the total sum of squares ratio of less than 1.25 (i.e., $\frac{\sum_t^{T-1}(f_{t+1}-f_t)^2}{\sum_t (f_t)^2} < 1.25$).

Starting with the 118,134 timecourses that had at least one point that passed the full noise model, we filtered - based on the timecourse-level patterns - 18,098 timecourses from further consideration, leaving 100,036 timecourses which contain clear impulse-like or sigmoidal dynamics.

3 Obtaining the *shrunken* gene expression dataset

We believe that each of the previously mentioned 100,036 timecourses contains timecourse-level signal, but not necessarily at every time point. Changes in the first 5 minutes are very rare (aside from the induced transcription factor), while near the end of the experiment, the vast majority of these timecourses will be different than their pre-induction expression level. To avoid the case of inappropriately interpreting early, often very weak expression variation as signal, we want to shrink signals to zero to the extent that they are consistent with the estimated noise-level of the gene $\hat{\sigma}^2_{ij}$. To determine whether each observation in a timecourse is consistent with the noise model, we perform a Wald test for every observation ($W_{ijt} = f_{ijt}/\hat{\sigma}_{ijt}$). As expected, Wald p-values at early timepoints are more uniformly distributed while later timepoints skew very strongly towards small p-values. This difference in the fraction of null hypotheses at a given time point (π_0) can be modeled as a monotonically decreasing function of time $\pi_0(t)$ using the functional false discovery rate [3, 4]. In order to make use of this approach for shrinkage estimation, we consider that each observation can be thought of as a mixture of two states, a null state where $\hat{f}_{ijt} = 0$ and an alternative state reflecting our experimental measurement: $\hat{f}_{ijt} = f_{ijt}$, weighted based on the relative support for an observation belonging to each state (w_{ijt}):

$$f_{ijt}^{\text{shrunken}} = w_{ijt} \times 0 + (1 - w_{ijt}) f_{ijt}^{\text{cleaned}}$$

w_{ijt} can be estimated across all the observation of a single timepoint (using the above estimated $\pi^0(t)$) using the local false discovery rate (LFDR) [4]. The LFDR is an estimate of the FDR of a single observation. Because π_0 is smaller for later timepoints, observations later in timecourses tend to be more similar to their measurements, while early timepoints are shrunk more aggressively towards zero. This is the "shrunken" dataset.

4 Obtaining kinetic parameters for timecourses

To compare the behavior of timecourses it is useful to be able to summarize both the timing and strength of transcriptional changes. To capture such simple dynamics, a sigmoidal model may often be sufficient:

$$y(t) \quad = \quad v_{\text{inter}} \frac{1}{(1 + \exp(-\text{rate} * (\text{time} - t_{\text{rise}})))}$$

However, in some cases the impulse model utilized above to define feasible timecourse-level signal may be necessary to capture complex dynamics:

$$y(t) \quad = \quad \frac{1}{1 + \exp(-\text{rate} * (\text{time} - t_{\text{rise}}))} * (v_{\text{final}} + (v_{\text{inter}} - v_{\text{final}}) * \frac{1}{1 + \exp(\text{rate} * (\text{time} - t_{\text{fall}}))})$$

In order to make use of kinetic information, we would like to determine when a sigmoidal versus an impulse model is supported and when and how strongly activations/inhibitions occur.

We are interested in generating interpretable parameters from such fits. Since sigmoidal or impulse models can fit the data in peculiar ways that are not biologically feasible (e.g., negative rate coefficients, impulses which fall before they rise, very strong late activations which are similarly captured with weaker activations), often with a similar fit to a reasonable parameterization, some constraints on parameters, through the use of priors, can greatly improve interpretability. To adapt this problem from non-linear least squares problem to one where we could apply prior constraints, sigmoid/impulse model parameters were estimated as maximum posterior (MAP) estimates ($\arg\max_{\Omega} \Pr(\Omega|\mathbf{y})$) over 100 initializations.

Gaussian likelihood defined the departures between observed fold changes and the non-linear prediction of an impulse or sigmoid model, while the parameters (Ω) of these predictions ($\hat{y}_i(t_i, \Omega)$) were constrained with Gaussian and Gamma priors:

$$
\begin{aligned}
\Pr(\Omega|\mathbf{y}) &\propto \Pr(\mathbf{y}|\Omega) \cdot \Pr(\Omega) \\
\Pr(\mathbf{y}|\Omega) &= \prod_{i=1}^{I} \mathcal{N}\left(\hat{y}_i(t_i, \Omega); \mu = y_i, \sigma = \hat{\sigma}_{ijt}\right) \\
\Pr(\Omega^{\text{sigmoid}}) &= \mathcal{N}(v_{\text{inter}}; 0, 1) \cdot \Gamma(\beta; 2, 0.25) \cdot \Gamma(t_{\text{rise}}; 2, 25) \quad \text{sigmoid} \\
\Pr(\Omega^{\text{impulse}}) &= \mathcal{N}(v_{\text{inter}}; 0, 1) \cdot \Gamma(\beta; 2, 0.25) \cdot \Gamma(t_{\text{rise}}; 2, 25) \cdot \\
&\quad \mathcal{N}(v_{\text{final}}; 0, 1) \cdot \Gamma(t_{\text{fall}} - t_{\text{rise}}; 2, 25) \quad \text{impulse}
\end{aligned}
$$

Each timecourse was fit with both a sigmoidal and an impulse model and we sought to determine which model best fit each timecourse. Since the sigmoidal model is a simpler, nested version of the impulse model (with $t_{\text{fall}} = \infty$), the likelihood ratio test was used to determine whether the sigmoid was significantly improved by the two extra parameters of the impulse model:

$$
\log \Pr(\mathbf{y}|\Omega^{\text{impulse}}) - \log \Pr(\mathbf{y}|\Omega^{\text{sigmoid}}) \sim \chi^2_2
$$

For $1,785$ timecourses, the impulse model fit significantly better than the sigmoid at Benjamini-Hochberg-based FDR of 0.001. These timecourses were said to have impulse dynamics while the remaining $98,251$ timecourses exhibited sigmoidal dynamics.

5 Dynamical systems modeling

We constructed a linear model based on a simple dynamical system model of genome-wide expression evolution. The time rate of change of a gene is modeled

as being affected by the expression levels of any of the genes in the genome, possibly linearly, or proportionally to the product of their expression levels. We can transform this equation into the same units as the cleaned data, with values at time zero divided out. We convert this into a regression problem by constructing a derivative estimator from the time series data. We then treat the left-hand side of the equation (i.e., the time derivative) as the dependent variable, modeled by the right-hand side (i.e., the linear and quadratic terms) as the independent variables.

$$\frac{d}{dt} z_i = \sum_j [A_{ij} z_j + B_{ij} z_i z_j] + D_i.$$

This system of equations presumes the natural units and reaction rates for gene expression. We only have the microarray measurements. We absorb the relation between transcript abundance and measured photons into the definitions of a new set of variables and a new set of coefficients and fit the normalized system. Our final variable y is the normalized ratio, y(t)=(r(t)/g(t))/(r(0)/g(0)), and y(t=0)=1 by definition.

$$\frac{d}{dt} y_i = \sum_j [\alpha_{ij} y_j + \beta_{ij} y_i y_j] + \delta_i.$$

The α matrix describes the linear effect of one gene's expression on another, while the β matrix describes the same effect when it is also proportional to the target gene's expression level. The δ vector represents a background level of transcription.

When an experiment begins, the yeast is approximately in a steady state. The population average gene expression should therefore be time independent at that point. We can subtract out this behavior explicitly, which would highlight any genes that violate this assumption. This σ vector explicitly describes the deviation from steady state at the start of the experiment.

$$\begin{aligned}
\sigma_i &= \sum_j [\alpha_{ij} + \beta_{ij}] + \delta_i, \\
\frac{d}{dt} y_i &= \sum_j [\alpha_{ij}(y_j - 1) + \beta_{ij}(y_i y_j - 1)] + \sigma_i.
\end{aligned}$$

Linear modeling of the gene expression levels can yield a negative numbers of transcripts which are clearly unphysical. If we instead model the log-ratios, this can never happen. Dividing each side by the time-dependent gene expression level $y_i(t)$ yields a new equation with the log derivative as the left-hand side. This equation is exactly equivalent, but notice that the source α, β terms and intercept σ terms now have the transcript ratio in the denominator.

$$\frac{d}{dt} \ln(y_i) = \sum_j \left[\alpha_{ij} \frac{y_j - 1}{y_i} + \beta_{ij} \frac{y_i y_j - 1}{y_i} \right] + \sigma_i \frac{1}{y_i}.$$

Enforcing the initial steady state means $\sigma_i = 0$, reducing to Eq. 1 in the main text.

We can invert the problem by simply integrating. Now, the left-hand side is the gene expression level, with the time zero subtracted. The right-hand side is the time integral over earlier time points. Thus, the expression level of a gene can be modeled by earlier-time averages of the expression levels of other genes.

$$\ln(y_i(t)) \quad = \quad \int_0^t dt' \left\{ \sum_j \left[\alpha_{ij} \frac{y_j(t') - 1}{y_i(t')} + \beta_{ij} \frac{y_i(t') y_j(t') - 1}{y_i(t')} \right] + \sigma_i \frac{1}{y_i(t')} \right\}.$$

6 Linear Regression

Translating the dynamical system described above into the language of linear regression is straightforward. In its simplest form, the dynamics of a single gene in a single intervention experiment is represented by a design matrix with rows corresponding to the (usually 8) time points and columns corresponding to the 6175 genes being measured. We first construct an estimator for the time derivative of the gene in question. This will reduce the effective number of independent microarrays by one per experiment. N points in a timecourse will provide N-1 derivative estimators. We choose to take the average of the first order forward and backward differences as our estimator, but note that this is not always the symmetric difference since the time samples are not uniform. Furthermore we assume that the backward difference at time zero is zero, and that the forward derivative at the end of the timecourse is also zero. However, we note that the N derivative estimates for the N time points are not linearly independent with these assumptions. Equivalently, if we consider a differencing operator that acts on a timecourse, applying it to a vector of 1s yields all zeros. Thus, the differencing operator is not invertible.

Leaving out the quadratic and intercept terms for clarity, the "derivative" and "integral" models can be written with the following equations, respectively:

$$\begin{aligned} \sum_Q D^{PQ} \ln(y_i^Q) \quad &\sim \quad \sum_j \alpha_{ij} \frac{y_j^P - 1}{y_i^P}, \\ \ln(y_i^P) \quad &\sim \quad \sum_{j,Q} \alpha_{ij} D^{-1,PQ} \frac{y_j^Q - 1}{y_i^Q}, \end{aligned}$$

where the P, Q indicices now refer to time.

The integral version models the data at a time point as a particular sum over earlier time points. In general, time sampling is not uniform. The majority of experiments are sampled at t = 0, 5, 10, 15, 20, 30, 45, 90 minutes. In this case, the difference and integral operators are, respectively:

$$D = \frac{1}{10 \text{ min}} \begin{pmatrix} -1 & 1 & 0 & 0 & 0 & 0 & 0 & 0 \\ -1 & 0 & 1 & 0 & 0 & 0 & 0 & 0 \\ 0 & -1 & 0 & 1 & 0 & 0 & 0 & 0 \\ 0 & 0 & -1 & 0 & 1 & 0 & 0 & 0 \\ 0 & 0 & 0 & -1 & 1/2 & 1/2 & 0 & 0 \\ 0 & 0 & 0 & 0 & -1/2 & 1/6 & 1/3 & 0 \\ 0 & 0 & 0 & 0 & 0 & -1/3 & 2/9 & 1/9 \\ 0 & 0 & 0 & 0 & 0 & 0 & -1/9 & 1/9 \end{pmatrix}$$

$$D^{-1} = 10 \text{ min} \begin{pmatrix} 0 & 0 & 0 & 0 & 0 & 0 & 0 & 0 \\ 1 & 0 & 0 & 0 & 0 & 0 & 0 & 0 \\ 0 & 1 & 0 & 0 & 0 & 0 & 0 & 0 \\ 1 & 0 & 1 & 0 & 0 & 0 & 0 & 0 \\ 0 & 1 & 0 & 1 & 0 & 0 & 0 & 0 \\ 2 & -1 & 2 & -1 & 2 & 0 & 0 & 0 \\ -1 & 2 & -1 & 2 & -1 & 3 & 0 & 0 \\ 8 & -7 & 8 & -7 & 8 & -6 & 9 & 0 \end{pmatrix}$$

The full regression dataset for the dynamics of the gene in question is constructed by row-wise stacking of the 200+ design matrices corresponding to the intervention experiments. We then repeat this for each of the 6175 genes.

7 BIC regularization

The previously explained regression formulation of the dynamic model was fit using lasso regression, and regularization paths were fit using glmnet [5].

Normally the regularization parameter of lasso regression is fit by straightforward cross-validation. Naively, we would use cross validation to select a λ for the dynamic model of each gene's dependence on other genes separately. However, there are a number of practical problems that make this inappropriate on this data.

First, although there are around ∼1600 rows in the design matrix that underlies the regression, these represent ∼200 experiments of 8 time points each. The point being that the interdependency of the data limits the kinds of cross-validation that are meaningful (e.g. ones in which whole time series are left out) and substantially reduces the effective sample size. In practice, separate cross validation for each gene's model is unacceptably unstable.

To explain our alternative approach, we make use of the theory, based on Stein's unbiased risk estimation approach, that the number of nonzero coefficients in lasso regression at a particular choice of λ is an unbiased estimate of the degrees of freedom of the model [6].

Accordingly, unbiased estimates of the BIC and AIC criteria can be formulated as $-2\log(L) + c\log(n)\widehat{df}$ where L is the likelihood of the data, $\widehat{df}$ is the number of nonzero coefficients in the model, and c is 1 or $2/\log(n)$, respectively. Note

that the dependence of L, $\hat{y}$, $\widehat{df}$, etc., on λ is suppressed in the notation, for brevity. Let sse $= ||y - \hat{y}||^2$. Then, for a homoscedastic Gaussian model, the deviance, D, i.e. -2 times the log-likelihood, follows:

$$D = \frac{\text{sse}}{\sigma^2} + n\log(\sigma^2)$$

where sse stands for the sum-squared errors of the model. Since D is minimized at $\hat{\sigma}^2 = \frac{\text{sse}}{n}$, we obtain:

$$\hat{D} = n\left(1 + \log\left(\frac{\text{sse}}{n}\right)\right)$$

This results in the following penalized criterion:

$$\text{criterion}_{v1} = n\left(1 + \log\left(\frac{\text{sse}}{n}\right)\right) + c\log(n)\widehat{df}$$

Rather than apply criterion_{v1} naively, we first address these concerns:

1. To be resilient to the small sample variability of the sse statistic (especially at small values)

2. To compensate for the fact that the homoscedastic noise model does not hold (most importantly because of time-dependence)

3. It is impractical to find a separate regularization parameter for each gene model separately by usual means, because the selections fluctuate too much.

To address the the first point, we can take advantage of the gene expression noise modeling, which gave us estimates of a noise level for each gene, τ_g. In relative units we can write $s^2 = (\text{sse}/n)/\tau_g^2$. Specifically, it is implausible for the predictive model to be able to explain the data substantially better than the τ_g ($s^2 < 1$) – these are likely to be due to chance.

We can rewrite $\hat{D}$ in terms of s^2 as: $\hat{D} = n(1 + \log(\text{sse}/n)) = n(1 + \log(s^2 * \tau_g^2)) = n\log(s^2) + n + 2n\log(\tau_g)$.

The second term is constant with respect to λ and may be omitted for model selection purposes. For the $n\log(s^2)$ term, however, by the above argument, this is a suitable functional form when s^2 is large, but not when s^2 is small. Various arguments can be made, but the form we finalized on was to replace $\log(s^2)$ with $\text{arcsinh}(s^2 - 1)$. This choice preserves the log-like behavior for large s^2, and the local linearity near 1, while giving a small maximum reward for the (probably chance) event that $s^2 < 1$. In summary the criterion we use for model selection is:

$$\text{criterion}_{v2} = n\ \text{arcsinh}(s^2 - 1) + c\log(n)\widehat{df}$$

Finally, we addressed points 2 and 3 by letting c be a tunable hyper-parameter whose value is shared across the dynamic expression models of all genes, and whose value will be chosen by a global cross-validation criterion.

8 Hyperparameters

For clarity, we enumerate the model choices (hyperparameters) that were evaluated. We started from "cleaned" dataset as described above.

- Data Preprocessing: Thresholding/Filtering/Gene+Chip noise model/LFDR
- Model formulation: Integral versus derivative
- Log vs. Linear: Dependent variable is the log derivative?
- Intercept: Allow the time derivative to be non-zero at t=0?
- Standardize: Covariates are scaled to unit normal before regression?
- Quadratic: Include quadratic independent variables?
- Regularization: Magnitude of the BIC adjustment

Data preprocessing is a discrete choice among thresholding levels. The next five are binary choices. The regularization level is, in principle, chosen from a continuum. In practice, we make a discrete selection among values separated by factors of 2, the factor 1.0 indicating the naive BIC calculation.

9 Model Validation by Holdout

Ideally, we would like to do a leave-one-out holdout analysis on the space of hyperparameters, at the transcription factor level. Since there are 200+ distinct transcription factor experiments (a few with replicates), and 6000+ genes, this involves computing 1.2M regression paths per choice of discrete hyperparameters. However, this is very computationally expensive. Therefore, we identified several transcription factors that remain active in the models out-of-sample. These are *CIN5, DAL80, FKH1, GAL4, GRX4, HAC1, HMS1, LEO1, MSN4, RDR1*, and *UGA3*. For this reduced set we exhaustively searched 128 choices of discrete hyperparameters. There are four binary choices (choosing not to standardize variables) and eight thresholding levels: none, LFDR, (zero, hard, soft thresholding) x (do continuity filtering). Including models with no holdouts, this corresponds to 12x128x6175 = 9.5M regularization paths computed. From these, we selected 34 candidates for full holdout runs (including checking a few where we do standardize variables). Over all hyperparameter and holdout selections, over 8000 regularization paths were generated for each transcript, over 50M in total.

To score a fully specified model (i.e. a complete specification of model hyperparameters) we used the method described in Algorithm 1. In order to score a holdout experiment, the model coefficients were interpreted as predictions about which genes would move up by 2-fold or down by 2-fold.

In ordinary cross validation, one tries to minimize out-of-sample squared-error. Instead, we choose to maximize out-of-sample coefficient correctness (i.e., the

coefficients' ability to make out-of-sample predictions about observed 2-fold changes). The models that scored best (on average) making out-of-sample predictions, including consideration of the sign (induction vs. repression), were called "best".

According to these criteria, the best performing model was:

- Data Preprocessing: zero thresholding, with continuity filter
- Derivative: dependent variable is the time derivative estimate
- Log: dependent variable is the log derivative
- No Intercept: prediction is forced to zero at time zero
- No Standardize: covariates are not whitened before regression
- Quadratic: quadratic terms are included in the design
- Regularization: BIC adjustment = 0.5

With these hyperparameters, we construct a final model with no experiments held out. The model takes the form of a number of coefficients stating that gene A induces or suppresses gene B.

10 Validation Experiments

Based on our modeling results, ten predicted latent regulators were chosen for experimental validation. Selected regulators fell into three classes of predictions:

- Regulators which were predicted to drive the strong impulse behavior of Pho4 induction experiment: Pho5, Pho11, Phm6
- Regulators predicted to affect many targets spanning all experiments: **Hmx1**, Arn2, Anb1, YGL117W
- Regulators predicted as hubs, operating in many experiments: **Stp4**, **Fmp48**, YGR066C

Each predicted regulator was separately induced and genome-wide expression was tracked at eight time points using the same experimental and computational methodology used to construct the "cleaned" dataset. Predicted regulators whose effects were primarily restricted to genes involved in the non-specific induction stress response (Phm6) were removed from consideration since their effects are either due to or confounded with the stress response. Predicted regulators were also removed from consideration if the induced gene increased by less than four-fold during the experiment (Pho11). For the remaining eight validation experiments, a regulator's predicted targets were compared to experimental changes (absolute fold-change > 0.5 at any time point) by constructing a contingency table and applying a χ^2-test to evaluate the independence of the marginal effects.

Algorithm 1 Hyperparameter search for dynamical systems modeling.

Require: raw data $\mathbf{D}_{\text{RAW}}$ ▷ (experiment, time) X gene
Require: gene set $G = \{g_i\}$ ▷ 6175 genes
Require: experiment set $H = \{h_j\}$ ▷ 200+ genes with direct experiments
Ensure: $H \subset G$
Require: hyperparameter sets $\Theta = \{\theta_k\}$ ▷ each θ_k is a collection of values
Require: BIC correction factors $\vec{B}$

for $\theta_k \in \Theta$ **do**
    $\mathbf{D} \leftarrow \textsc{PreprocessData}(\mathbf{D}_{\text{RAW}}, \theta_k)$
    for $h_j \in H$ **do**
        $\mathbf{D}_H \leftarrow \textsc{HoldOutExperiment}(\mathbf{D}, h_j)$
        $\{\vec{\beta}_i(\vec{B})\} \leftarrow \textsc{FitModel}(\mathbf{D}_H, \theta_k, \vec{B})$
        $\mathbf{D}_K \leftarrow \textsc{KeepOnlyExperiment}(\mathbf{D}, h_j)$
        $S_j(\vec{B}) \leftarrow \textsc{ScoreModelCoefficients}(\{\vec{\beta}_i(\vec{B})\}, \mathbf{D}_K)$
    end for
    $\Omega(\theta_k, \vec{B}) \leftarrow \textsc{CollateHoldoutScores}(\{S_j(\vec{B})\})$
end for
$\theta, B \leftarrow \text{argmax } \Omega(\Theta, \vec{B})$
$\mathbf{D} \leftarrow \textsc{PreprocessData}(\mathbf{D}_{\text{RAW}}, \theta)$
$\{\vec{\beta}_i^{\text{FINAL}}\} \leftarrow \textsc{FitModel}(\mathbf{D}, \theta, B)$

procedure $\textsc{FitModel}(\mathbf{D}_{\text{in}}, \theta_k, \vec{B})$
    for $g_i \in G$ **do**
        if $g_i \in H$ **then**
            $\mathbf{D} \leftarrow \textsc{HoldOutExperiment}(\mathbf{D}_{\text{in}}, g_i)$ ▷ g_i experiment held out
        else
            $\mathbf{D} \leftarrow \mathbf{D}_{\text{in}}$
        end if
        $\mathbf{X} \leftarrow \textsc{ConstructDesignMatrix}(\mathbf{D}, g_i, \theta_k)$
        $\vec{y} \leftarrow \textsc{ConstructDependentVariable}(\mathbf{D}, g_i, \theta_k)$
        $\vec{\beta}_i(\vec{\lambda}) \leftarrow \textsc{FitLASSO}(\mathbf{X}, \vec{y}, \theta_k)$ ▷ model is $\vec{y} \sim \mathbf{X}\vec{\beta}$
        $\vec{\beta}_i(\vec{B}) \leftarrow \text{BIC\_}\textsc{CorrectionFactor}(\mathbf{X}, \vec{y}, \vec{\beta}_i(\vec{\lambda}))$ ▷ $\vec{\lambda} \rightarrow \text{BIC}$
    end for
    return $\{\vec{\beta}_i(\vec{B})\}$
end procedure

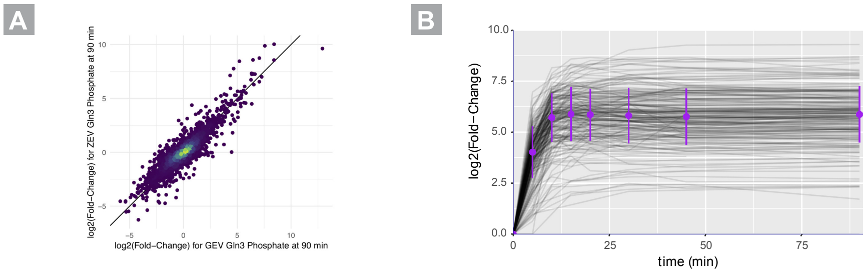

Appendix Figure S1: Gene expression responses do not depend on triggering mechanism and TF induction is rapid across our dataset. (A) Scatterplot of gene expression responses following GLN3 induction (using the "cleaned" dataset) at 90 min using either GEV or ZEV. (B) The expression level of each TF is plotted from the experiment in which the TF is induced with 1 μM β-estradiol (grey). The median expression level across all TF inductions (purple) at t = 0, 5, 10, 15, 20, 30, 45, and 90 minutes following induction is also shown (the errorbar width is ±1 standard deviation).

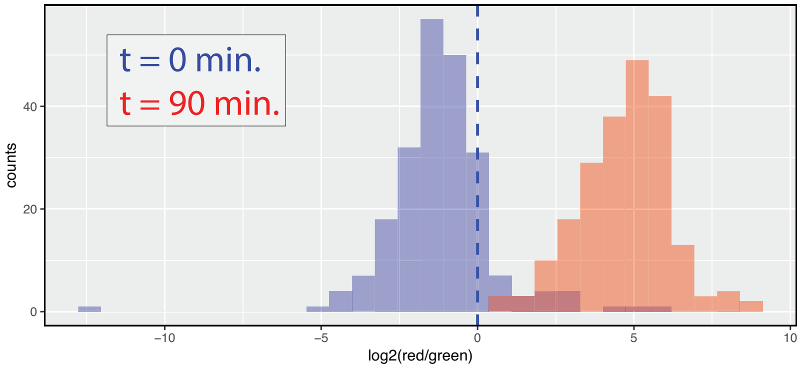

Appendix Figure S2: Comparing expression of synthetic promoter-driven TF alleles to native promoter-driven TF alleles. For each TF strain, the red (sample) and green (reference) microarray values were obtained from the t = 0 min. and t = 90 min. samples. The red/green ratio provides an estimate of leakiness for the t = 0 min. histogram (blue) in which 86% of synthetic promoter-driven TFs have expression less than WT TF levels. At t = 90 min., the red/green ratio provides an estimate of induction above WT TF levels (red histogram). The median level of TF induction over WT TF levels is 28.4.

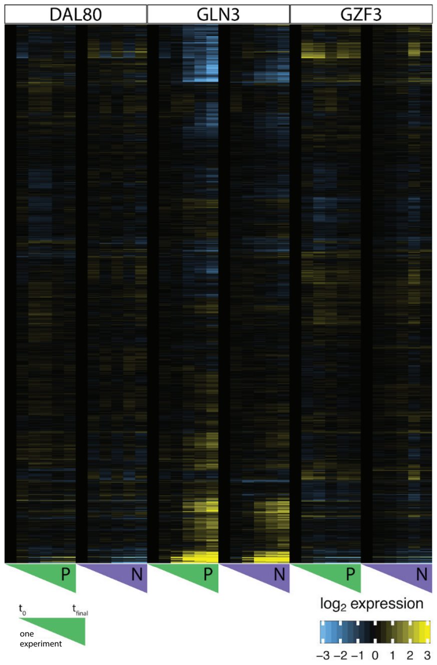

Appendix Figure S3: Induced changes can be robust to environmental state. Three regulators that were separately induced in either a phosphorus- or nitrogen-limited environment and the resulting "cleaned" experiments are shown. From the "shrunken" data, the correlations of the paired experiments are 0.40, 0.87, and 0.77 for Dal80, Gln3, and Gzf3, respectively.

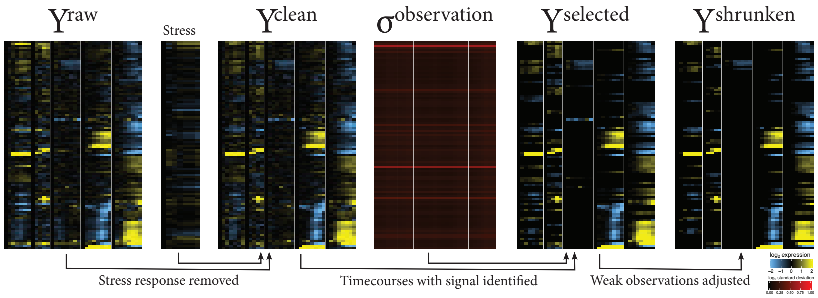

Appendix Figure S4: Systematic identification and summarization of regulatory signals. Since most transcriptional regulators affect a relatively small number of target genes, meaningful changes in expression are relatively sparse (9% of timecourses). These signal-containing timecourses are distinguished from timecourses which are purely noise, by first regressing out an average stress response, then selecting timecourses with extreme observation-level signal-to-noise and finally shrinking observations towards zero based on signal-to-noise.

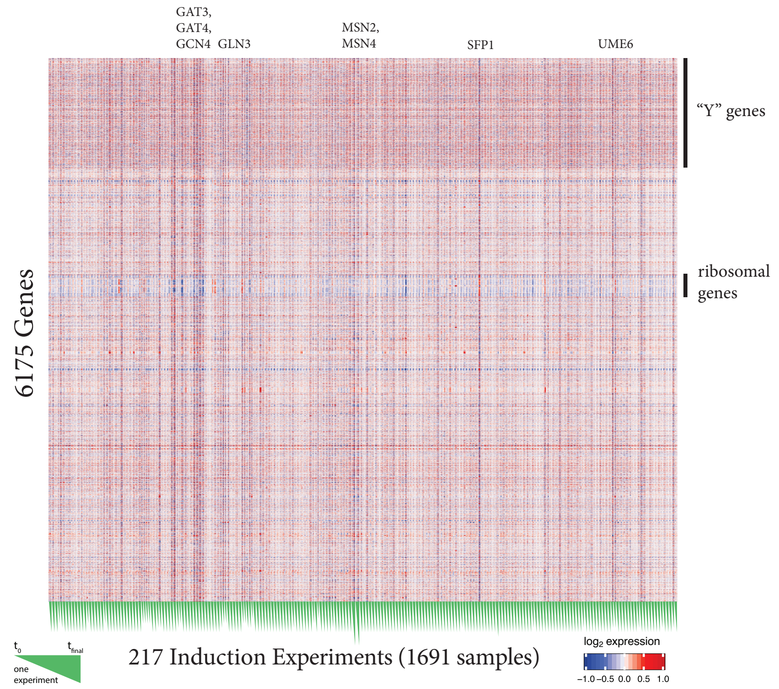

Appendix Figure S5: Full transcriptome of the "raw" gene expression data. Genes are sorted alphabetically from bottom to top. Note the increase in variability of gene expression for genes near the top of the clustergram (these are mostly genes that begin with "Y" as they have no known function and lack a standard name). There is also a clear horizontal stripe for the ribosomal genes, which are weakly repressed in many experiments as part of a mild stress response. The appearance of vertical stripes in the heatmap is due to TFs with many regulatory connections (hubs), a few of which are highlighted including: GAT3, GAT4, GCN4, MSN2, MSN4, SFP1, and UME6.

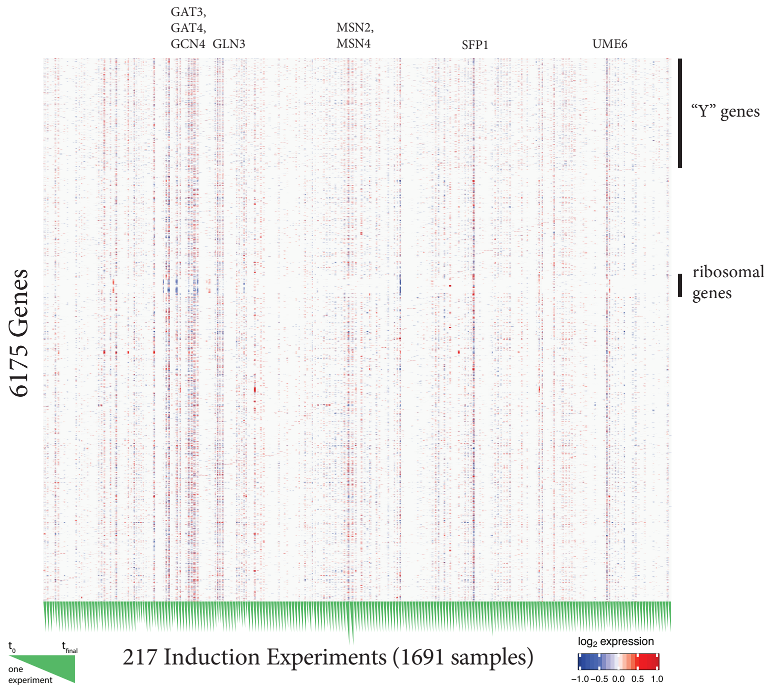

Appendix Figure S6: Full transcriptome of the "shrunken" gene expression data. Genes are sorted alphabetically from bottom to top. Vertical stripes in the heatmap are from TFs with many regulatory connections (hubs). A subset of these are highlighted: GAT3, GAT4, GCN4, MSN2, MSN4, SFP1, and UME6. Data cleaning removes noise from "Y" and ribosomal genes (seen in Appendix Figure S5) to reveal remaining signal.

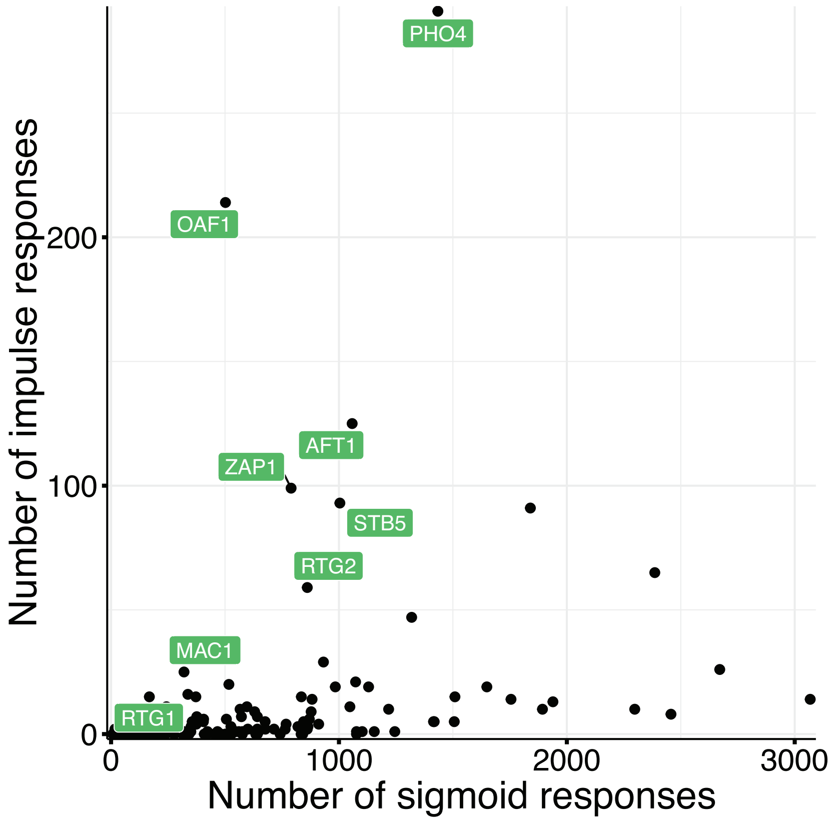

Appendix Figure S7: Counts of impulse vs. sigmoids across experiments. Transcriptional regulators are characterized based on the number of transcriptional responses that are sigmoid (e.g., turn on) versus impulses (e.g., turn on, then off). Pho4, Oaf1, Aft1, and Zap1, among other transcription factors, are highly enriched for impulse dynamics. Impulses generally feature spikes in enzyme expression and dips in ribosome synthesis.

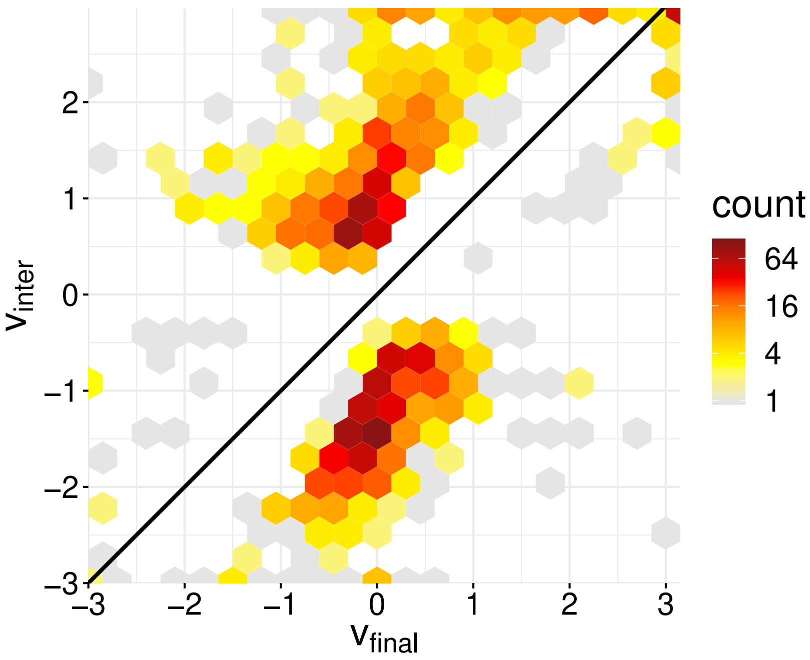

Appendix Figure S8: Most impulses exhibit near-perfect adaptation. v_{inter} **is compared to** v_{final} **for all timecourses exhibiting impulse dynamics.** The absolute value of each coefficient was floored to three for visualization.

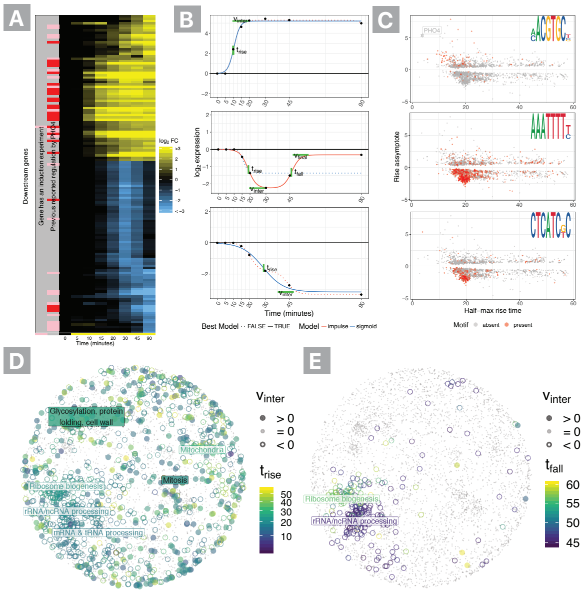

Appendix Figure S9. Diverse, functionally significant regulation in the Pho4 induction experiment. A. Heatmap summary of Pho4 induction experiment showing all > 4-fold changes. B. Parametric summaries of representative sigmoidal activation/inhibition timecourses and impulse (double sigmoid) modeling of transitory inhibition. Sigmoids are summarized by half-max time (t_{rise}) and the asymptote (v_{inter}), while impulses include a second half-max (t_{fall}) time and final asymptote (v_{final}). The strongest supported model for each timecourse is shown as a filled in line, while the alternative model is shown with a dashed line. C. K-mers enriched in the promoters of regulated genes are overlaid on summary of each gene's t_{rise} and v_{inter}. D. Response kinetics are overlaid on gene coordinates based on synthetic lethality as a surrogate for functional similarity. Pho4 rapidly inhibits ribosome biogenesis, rRNA processing and mRNA processing. E. The rRNA processing and ribosome biogenesis responses are each acute inhibition impulses that can be clearly distinguishing based on kinetics.

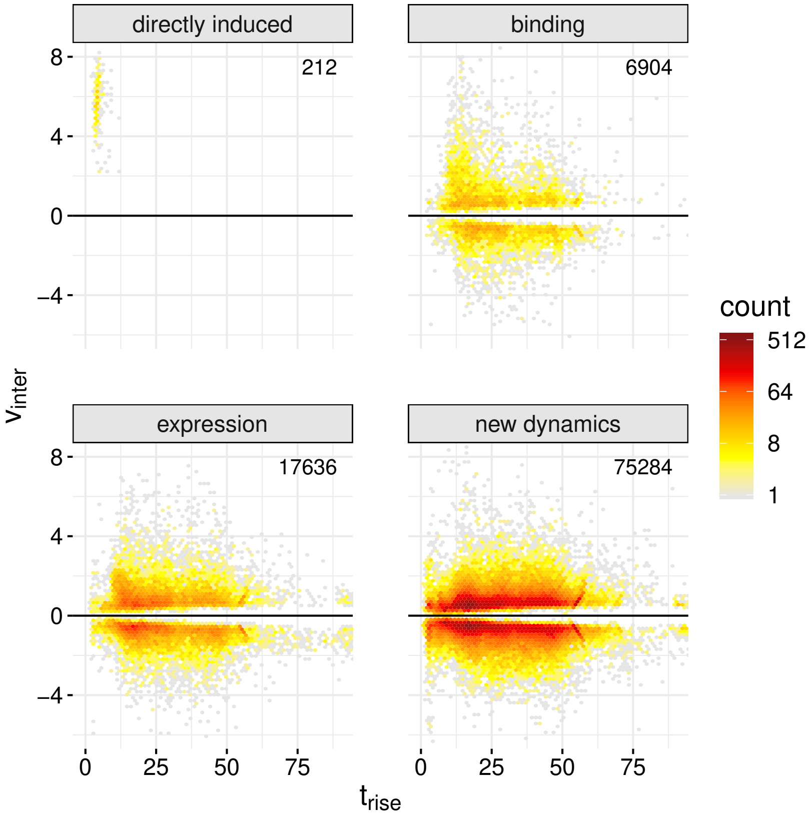

Appendix Figure S10: Functional classes of kinetic responses. For each of the $> 100,000$ timecourses with parametric fits, timecourses were divided into four categories: directly induced (TF induced in cognate experiment), binding (direct regulation based on direct-binding data from Yeastract), expression (co-expression of TF and responsive gene based on data from Yeastract), and new dynamics (newly discovered gene associations outside the other three classes).

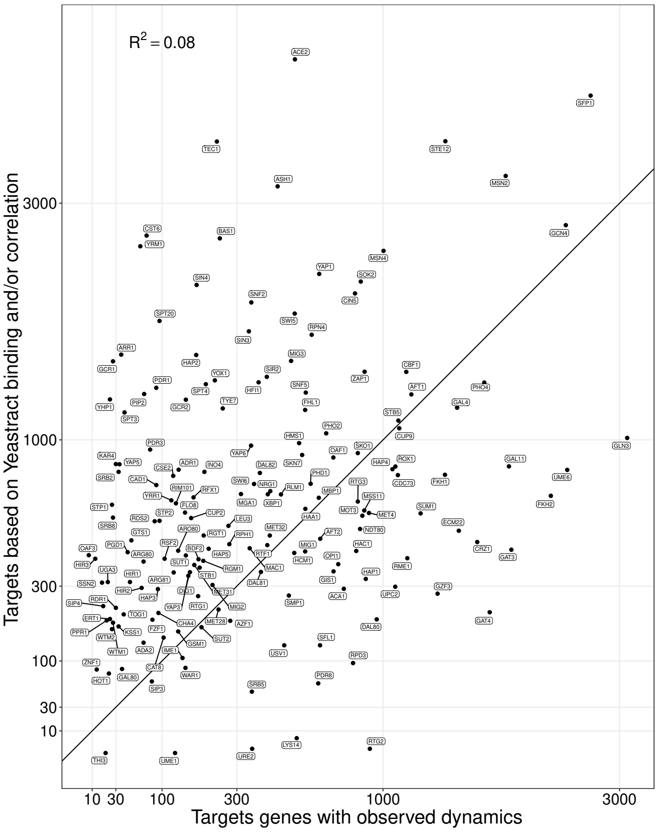

Appendix Figure S11: Response magnitude comparison with reported regulation. Scatter plot of number of targets or correlation-associated genes (Yeastract) versus the number of genes with significant dynamical responses in the "shrunken" data.

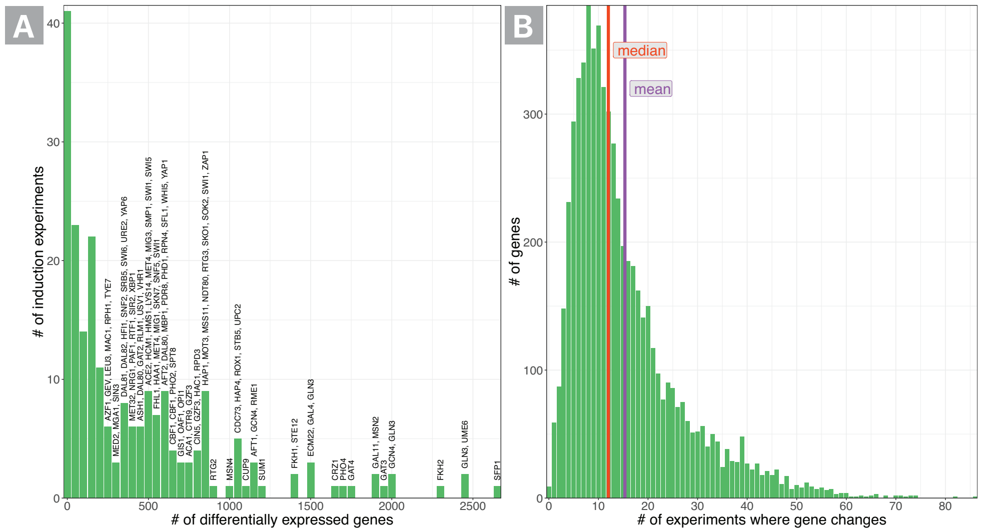

Appendix Figure S12: Extent of differential expression per experiment or gene. A. Histogram of the number of differentially expressed genes in each experiment. B. Histogram of the number of transcriptional regulators under which a gene changes (median 12, mean = 15.3, maximum would be changing under all 203 distinct induction experiments). Regulators resulting in differential expression of less than 50 genes include ARR1, CAM1, ERT1, FAP1, GAL80, GCR1, GTS1, HIR1, HIR3, HMRA1, HOT1, KAR4, KSS1, LOT6, MTF1, OAF3, PGD1, PPR1, RDR1, RDS2, RRN10, SIP4, SPT3, SRB2, SRB8, SSN2, STP1, TAF2, THI3, TOG1, UAF30, UGA3, WTM1, WTM2, YAP5, YHP1, YLL054C, and ZNF1.

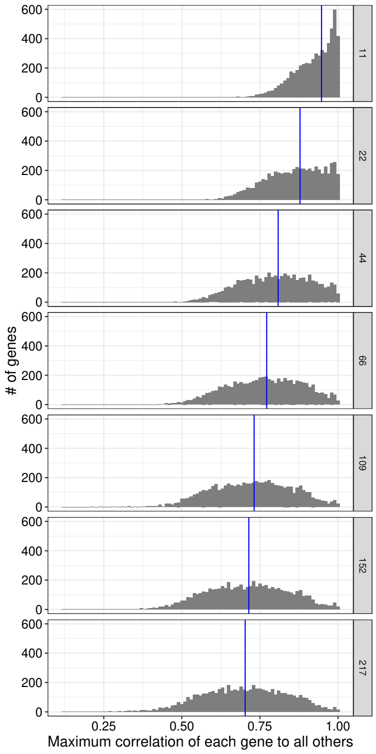

Appendix Figure S13: Histograms of maximum correlation of genes to all other measured genes are shown as experiments are pruned from the dataset. Panels indicate the number of experiments included in the analysis, where smaller datasets retain the experiments with the largest number of differentially expressed genes. Each gene is summarized based on the maximum correlation of its expression across all included experiments and timepoints to every other genes' expression. The blue line indicates the median of the maximum correlation of genes. For the purpose of calculating medians, genes which are dropped when constructing the reduced datasets are represented with a correlation of one (since they are impossible to discriminate from other absent regulators).

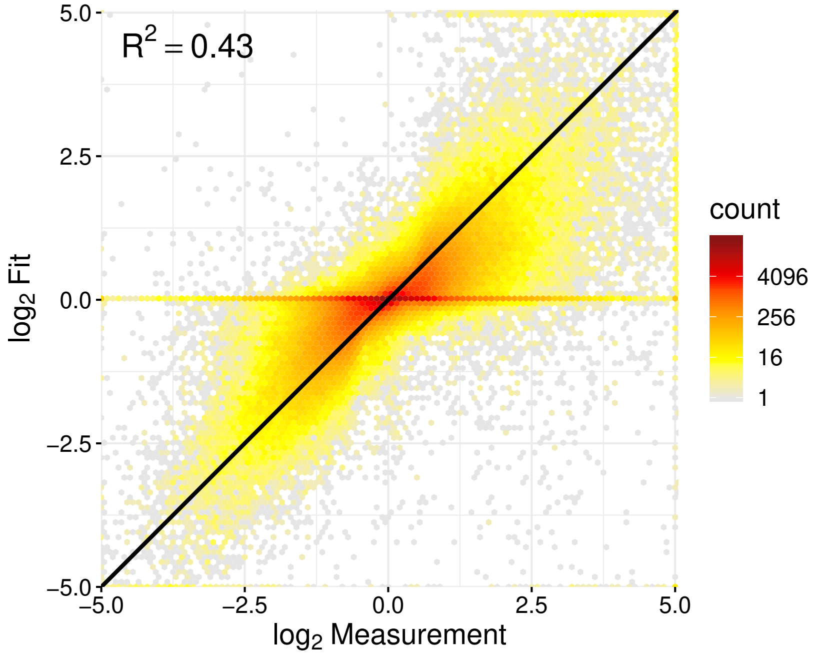

Appendix Figure S14: Fit of regulatory model to observed gene-expression measurements. Each observation compares measured log2 fold-changes from the dataset that the whole-cell model was fit to (i.e., time courses that passed full noise model and filters; see section 8 of the supplement) with the fitted fold-changes predicted from the whole-cell model.

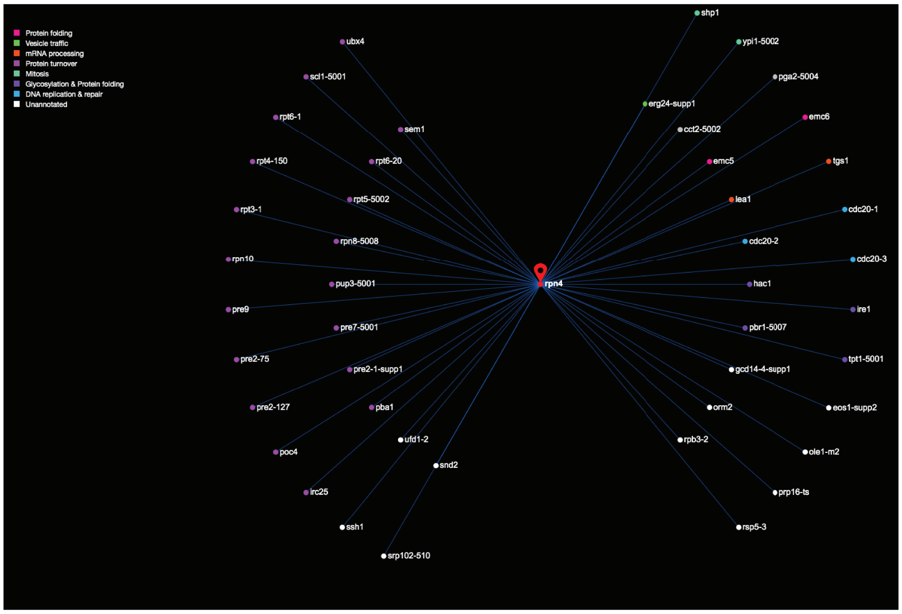

Appendix Figure S15: Negative genetic interactions with rpn4Δ from http://thecellmap.org. Strong negative genetic interactions are shown (interaction score < -0.45). Genes are colored according to SAFE annotations.

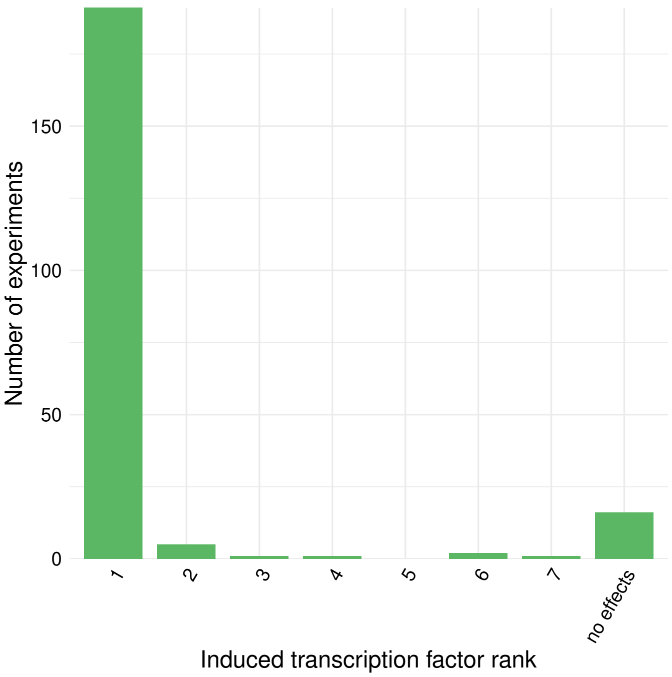

Appendix Figure S16: Induced transcriptional regulators are the primary drivers of gene expression changes in most experiments. Each experiment summarized differentially expressed genes based on the regulator with the largest attributed role in achieving the rise time. The rank of the induced transcription factor among regulators, derived from the model, is shown across all experiments. TFs that were not predicted by the model to directly drive changes in their respective experiments: DAL80, GAL4, HAP3, HMRA1, LOT6, MTF1, RRN10, RSF2, SIP3, TFB5, WHI5, and ZNF1.

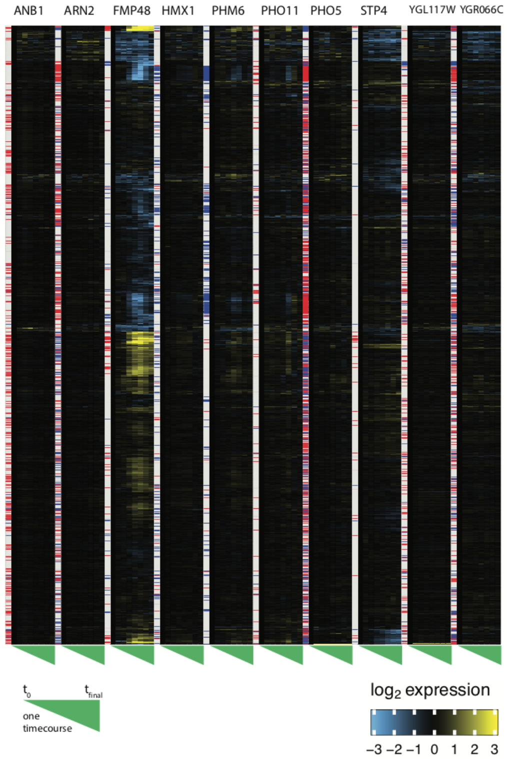

Appendix Figure S17: Transcriptome-wide heatmap for 10 validation experiments. Model-predicted regulatory targets of each putative regulator are shown to the left of time zero with predicted activation shown in red and predicted inhibition shown in blue.

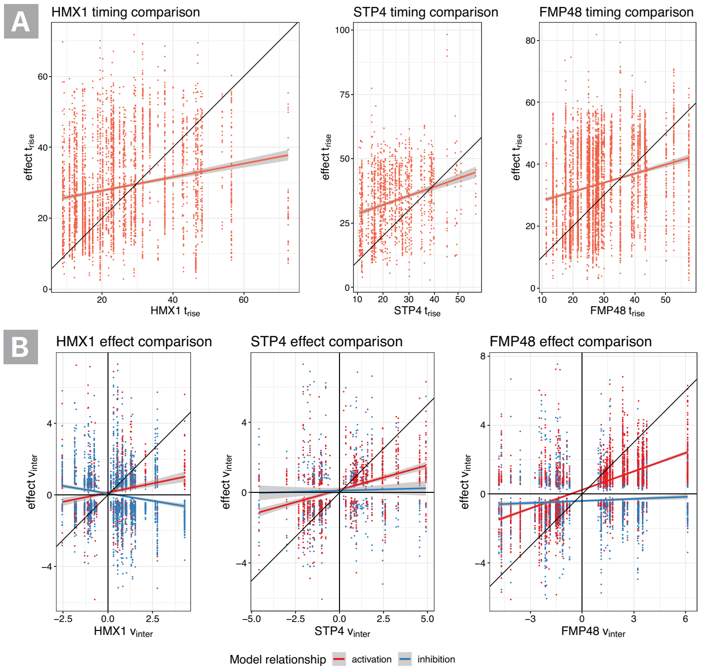

Appendix Figure S18: Identifying latent transcriptional regulatory hubs. A. For each gene whose expression change is partially attributed (attributing at least 5% of variation) to a regulator's levels in 5 or more experiments, the timing of the regulator is compared to its predicted targets within the same experiment. B. The v_{inter} value (i.e., expression-level asymptote) of each regulator is compared to the v_{inter} of each of its effects in the same experiment. Targets are colored based on whether the regression model indicates an activating or inhibitory relationship.

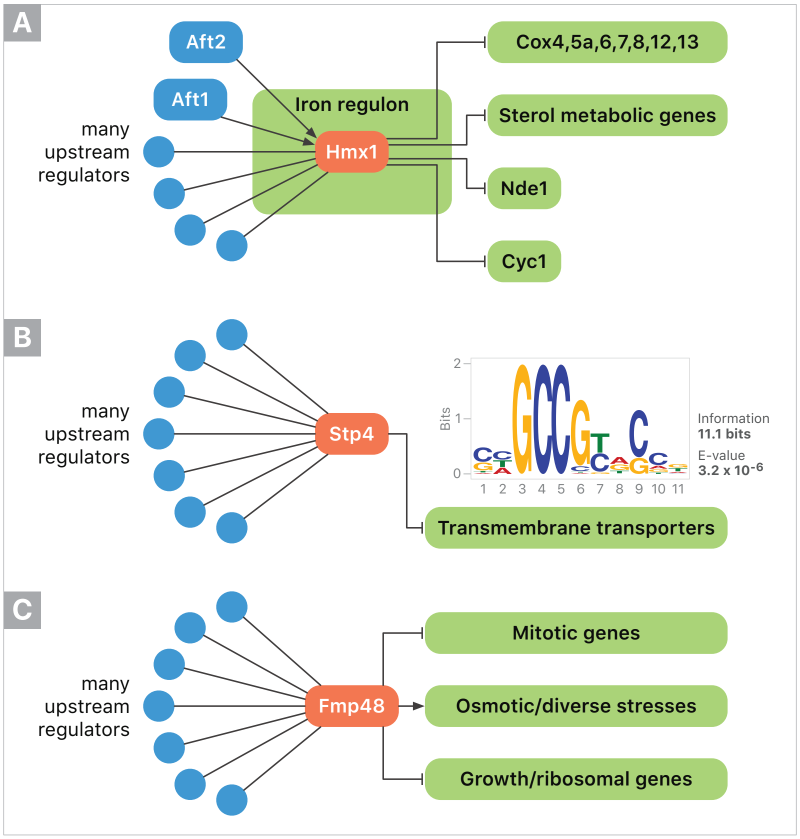

Appendix Figure S19: GRNs. TFs with induction experiments used for modeling are shown in blue. Genes that were induced based on model predictions are shown in orange, and responses to those genes are shown in green for A. Hmx1, B. Stp4, and C. Fmp48.

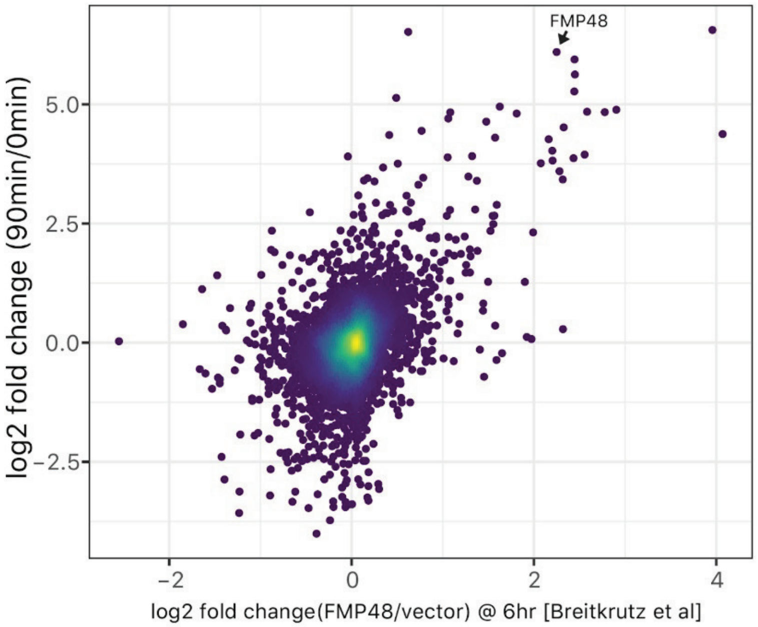

Appendix Figure S20: Comparing transcriptome data from two Fmp48 overexpression datasets. Data shown in Figure 5 are plotted along the y-axis. Replicates from Breitkreutz et al. (2009) are averaged and plotted along the x-axis. The Pearson correlation is 0.47 and is significant with p-value $< 2.2 \times 10^{-16}$.

References

[1] Donoho DL, Johnstone IM, Kerkyacharian G, Picard D. *Wavelet shrinkage: asymptopia?* Journal of the Royal Statistical Society Series B. 1995; 57(2):301-369.

[2] Chechik G, Koller D. *Timing of gene expression responses to environmental changes.* J Comput Biol. 2009 Feb;16(2):279-90.

[3] Chen X, Robinson DG, Storey JD *The Functional False Discovery Rate with Applications to Genomics.* bioRxiv 241133; doi: https://doi.org/10.1101/241133.

[4] Storey JD, Tibshirani R. *Statistical significance for genomewide studies.* PNAS. 2003. 100(16):9440-5.

[5] Friedman, J., Hastie, T., Tibshirani, R. *Regularization paths for generalized linear models via coordinate descent.* Journal of Statistical Software 2010, 33(1):1.

[6] Zou, H., Hastie, T., Tibshirani, R. et al. *On the degrees of freedom of the lasso.* The Annals of Statistics. 2007; 35(5): 2173-2192.
